# Improving Rural Access to Urological Healthcare: Patient Experiences With Cystectomy and Urinary Diversion

**DOI:** 10.1111/iju.70033

**Published:** 2025-03-04

**Authors:** Erica Zeng, Megan Saucke, Bhabna Pati, Alexa Rose, Esra Alagoz, Kyle A. Richards

**Affiliations:** ^1^ Department of Urology The University of Wisconsin School of Medicine and Public Health Madison Wisconsin USA; ^2^ Department of Surgery The Wisconsin Surgical Outcomes Research Program Madison Wisconsin USA

**Keywords:** bladder cancer, cystectomy, quality improvements, urinary bladder neoplasms, urinary diversions

1

Patients with bladder cancer (BCa) face multiple challenges from diagnosis to treatment. Assessments of rural versus urban location have resulted in mixed findings on outcome disparities [1, 2]. However, with increasing numbers of rural Americans and concurrent urological workforce shortages, particularly in those settings, rurality's impact on health is increasingly relevant [3]. Due to these concerning shifts, along with the inherent risks associated with BCa and cystectomy, we sought to utilize patient engagement to identify areas of improvement for care within the rural setting of Wisconsin (WI) state.

We conducted 5 focus groups of patients with BCa treated with cystectomy and urinary diversion (*n* = 17) at the University of Wisconsin (UW):

1. Women with ileal conduit (*n* = 3).
2. Women with continent diversion (*n* = 3).
3. Men with continent diversion (*n* = 3).
4. Men with ileal conduit (*n* = 3).
5. Men with ileal conduit (*n* = 5).

A standardized script was utilized, and each session was recorded and transcribed verbatim. Transcripts were assessed using the *Sort and Sift*, *Think and Shift* method, where the data was explored with frequent steps back to assess content and context, with insights directing the creation of a flexible codebook in NVivo, a qualitative analysis software program [4]. Thematic summaries were drawn from individual codes to characterize salient findings.

Participants had varying experiences outside of UW (Figure 1a). Most found that while some BCa treatments (e.g., chemotherapies, simple resection) were available in other WI regions, UW offered more surgical options. Certain cystectomy approaches and diversion types were not performed by local surgeons. Some patients also felt that they were given incomplete information regarding available treatment options. This was upsetting to some, since they had to depend on their own research to get more complete information. Patients reported mixed experiences when dealing with doctors other than their UW surgeon. One liked that their local long‐term provider shared input based on familiarity with the patient's lifestyle. Some found it helpful to use information they learned from the UW Surgeon to get a second opinion. Others felt frustrated by their experiences with other providers and said that their UW provider usually provided more information. The UW provider sends letters to local urologists to aid in communication.

**FIGURE 1:**
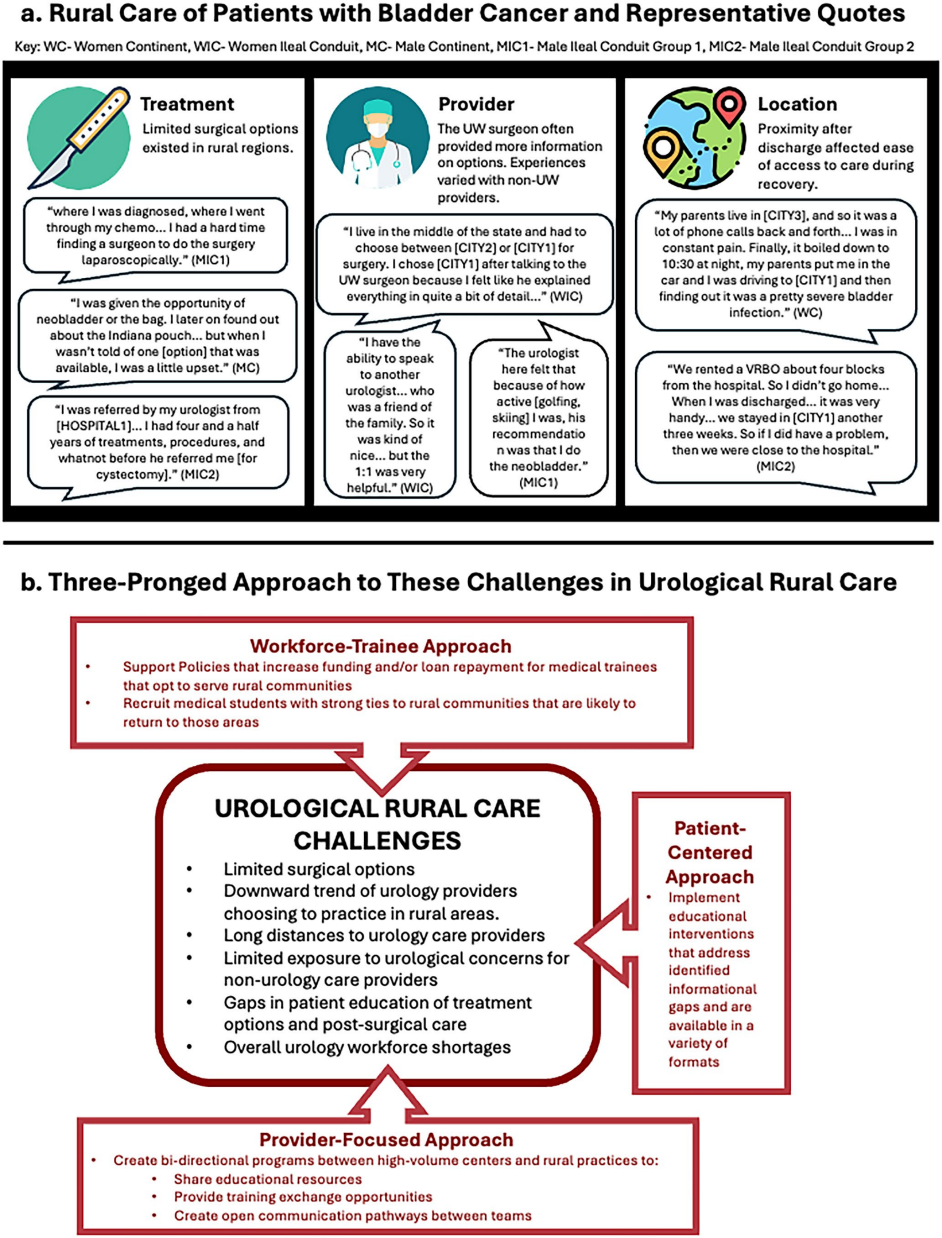
Patient quotes reflecting key themes experienced during rural urological care and proposed approaches to addressing these concerns. (a) Rural care of patients with bladder cancer and representative quotes. (b) Three‐pronged approach to these challenges in urological rural care.

Locations after discharge affected how easily patients could access surgical aftercare. One patient who lived 3 h away rented housing near the hospital after discharge, which made it convenient to receive postoperative care. A patient recovering farther away described care team interactions via phone rather than in‐person evaluations and then needed to drive a significant distance to UW when the surgeon's assessment from afar was inaccurate. Some patients experienced delayed responses from the surgical team to questions, and others dealt with suboptimal processes for accessing care in person (e.g., exposure to emergency care settings, interactions with providers unfamiliar with their surgeries). Home health care was often cited as a suitable bridge to care access, particularly for non‐emergent concerns.

Patients described challenging experiences with BCa in rural WI, including lack of access to complete information, limited surgical options, and trouble accessing timely follow‐up care after surgery. Importantly, these findings can be applied to urological rural care beyond WI and throughout the United States. We suggest a three‐pronged approach to addressing these specific challenges (Figure 1b). The first prong is improving patient‐facing educational materials to address informational shortcomings. The second is creating a bi‐directional exchange program to expand outreach clinics, which have been effective in addressing urology shortages [5]. Creating a reciprocal relationship between high‐volume urology centers and rural programs provides training opportunities for those wanting additional clinical exposure, limits travel burdens on single parties, and enables resource sharing regarding surgical options, urinary diversion care, and patient education. The third prong is addressing workforce/trainee constraints by supporting efforts to increase the number of both urology and rural practitioners [6]. These suggestions may also be broadly applicable to any surgical subspecialty concerned with disparities affecting their rural patients. This work is limited by sampling bias and coming from a single center.

## Author Contributions

**Erica Zeng:** investigation, writing – original draft, methodology, validation, visualization, writing – review and editing, formal analysis, data curation. **Megan Saucke:** conceptualization, investigation, writing – review and editing, writing – original draft, validation, methodology, formal analysis, project administration, data curation. **Bhabna Pati:** conceptualization, investigation, writing – review and editing, validation, methodology, formal analysis, data curation. **Alexa Rose:** conceptualization, investigation, funding acquisition, methodology, validation, formal analysis, data curation. **Esra Alagoz:** supervision, formal analysis, project administration, validation, methodology, writing – review and editing, investigation. **Kyle A. Richards:** investigation, funding acquisition, conceptualization, writing – review and editing, visualization, validation, methodology, project administration, supervision, formal analysis, data curation, resources.

## Ethics Statement

Approval of the research protocol by an Institutional Reviewer Board: N/A.

Registry and the Registration No. of the study/trial: N/A.

Animal Studies: N/A.

## Consent

The authors have nothing to report.

## Conflicts of Interest

The authors declare no conflicts of interest.
